# Flexible electrical aptasensor using dielectrophoretic assembly of graphene oxide and its subsequent reduction for cardiac biomarker detection

**DOI:** 10.1038/s41598-019-42506-1

**Published:** 2019-04-12

**Authors:** Abhinav Sharma, Jaesung Jang

**Affiliations:** 1 School of Materials Science and Engineering, Ulsan National Institute of Science and Technology (UNIST), Ulsan, 44919 South Korea; 2 School of Mechanical, Aerospace and Nuclear Engineering, UNIST, Ulsan, 44919 South Korea; 3 Department of Biomedical Engineering, UNIST, Ulsan, 44919 South Korea

## Abstract

Cardiac troponin T (cTnT) is considered a clinical standard for its high specificity and sensitivity when diagnosing acute myocardial infarction; however, most studies on the electrical sensors of cardiac troponin biomarkers have focused on cTnI rather than cTnT. This study presents label-free, low-cost, transparent, and flexible aptamer-based immunosensors for the electrical detection of cTnT using reduced graphene oxide (rGO) sheets. GO was first deposited by AC dielectrophoresis between two predefined source and drain electrodes on a 3-aminopropyltriethoxylsilane-modified polyethylene terephthalate substrate. The GO was then reduced using hydrazine vapour without damaging the substrate, resulting in uniform, controlled, and stable deposition of rGO sheets, and demonstrating more stability than those directly deposited by dielectrophoresis. Amine-modified single-strand DNA aptamers against cTnT were immobilized onto the rGO channels. The relative resistance change of this sensor owing to the attachment of cTnT was quantified as the cTnT concentration decreased from 10 ng/mL to 1 pg/mL in phosphate buffered saline (PBS) and 10-fold diluted human serum in PBS, with the limits of detection being 1.2 pg/mL and 1.7 pg/mL, respectively, which is sufficiently sensitive for clinical applications. High-yield and rapid fabrication of the present rGO sensors will have significant influences on scaled-up fabrication of graphene-based sensors.

## Introduction

Cardiovascular disease is one of leading causes of mortality and morbidity globally. The statistics based on World Health Organization (WHO) showed that 17.9 million deaths were attributed to this disease in 2015^1^, with 7.3 million being due to acute myocardial infarction (AMI)^2^. Among several biomarkers for the detection of AMI, both cardiac troponins (I & T) are considered the “gold standard” owing to their high sensitivity and specificity for cardiac muscle damage^3^. A complex of cardiac troponins comprises cardiac troponin I (cTnI), cTnT, and cTnC, which are found in cardiac muscles, and both cTnI and cTnT are highly specific. In clinical practice, cTnT values provide an accurate diagnosis of absolute infarct size in AMI^4^. After the onset of AMI, the concentrations of the cardiac troponins begin to rise within 4–6 h, elevated up to more than two weeks for cTnT and more than 5–7 days for cTnI^5^. In human serum, cTnT values <0.05 ng mL^−1^ are considered normal for AMI, values of 0.05–0.09 ng mL^−1^ are considered borderline, and values ≥0.1 ng mL^−1^ are considered positive^6^. Despite the widespread use of cTnT and cTnI as diagnostic tools for AMI, commercially available cTnI immunoassay kits show large variations (at least a 5-fold difference) in the measured concentrations among them^7^. Moreover, complex forms of cTnI and its low stability could be problematic for developing new analytical methods. Therefore, cTnT assays may be more reliable for wider applications^8,9^.

Conventional immunoassays employed for the detection of cTnT include enzyme-linked immunosorbent assay^10^, fluoroimmunoassay^11^, radioimmunoassay^12^, immunochromatographic tests^13^, and electrochemiluminescence immunoassay^14^. However, these assays are usually time-consuming, expensive, and need multi-step processing of samples and good skilled staff.

In this regard, the development of low-cost, portable and sensitive biosensors is of significant interest for disease prognosis and diagnosis in hospitals and for point-of-care testing (POCT) applications^15,16^. Several immunosensors have been presented for cTnT detection using different principles, such as surface plasmon resonance, electrochemical principles, etc. (Table 1). Although electrical immunosensors for cTnT detection have received little attention^17–19^, electrical sensors generally show several advantages, including simple measurements, easy miniaturization, and no necessity for complex instrumentation units^20^. Therefore, fabrication of electrical sensors on low-cost and flexible substrates would be highly desirable for POCT applications.

**Table 1:** Various types of immunosensors for detection of cardiac Troponin T.

Sensor platform/Materials	Sensor type	Recognition element/substrate	Media	Measurement range/Detection limit	References
Nanostructured CNTs-PEI on an AuE	Electrochemical (CV)	Antibody/AuE	PBS, Serum	0.1–10 ng mL^−1^ (PBS) LOD–0.033 ng mL^−1^ 0.02–0.32 ng mL^−1^ (Serum)	^58^
Polyaniline derivative poly-*o*-ABA modified electrode	Electrochemical (Chronoamperometry)	Antibody/GCE	PBS, Serum	0.05–10 ng mL^−1^ (PBS) LOD–0.016 ng mL^−1^ 0.025–7.5 ng mL^−1^ (Serum) LOD–0.088 ng mL^−1^	^59^
MWCNTs modified with artificial Abs	Electrochemical (Potentiometry)	MIP	Serum	1.41–20.86 µg mL^−1^ LOD–0.16 µg mL^−1^	^9^
N-MIP with co-polymer matrix rGO electrode surface	Electrochemical (DPV)	MIP/SPE	PBS, Serum	0.01–0.5 ng mL^−1^ (PBS) LOD–0.006 ng mL^−1^ 0.017–0.28 ng mL^−1^ (Serum)	^60^
Nanostructured ZnO electrodes	Electrochemical (EIS)	Antibody/Polyimide	Serum	0.0001 ng mL^−1^–100 ng mL^−1^ LOD–1 pg mL^−1^ (Serum)	^61^
Two planar Al electrodes	Capacitance measurement	Antibody/SiO_2_/Si	PBS, Serum	0.01–5 ng mL^−1^ (PBS), 0.07–6.83 ng mL^−1^ (Serum)	^62^
Gold substrate functionalized with SAM layer	SPR	Antibody/AuE	PBS	0.1–50 µg mL^−1^ LOD–100 ng mL^−1^	^63^
Sandwich immunoassay with AuNPs	Fluorescence	Antibody	Serum	0.25–14 nM LOD–0.02 nM (0.7 ng mL^−1^)	^11^
cTnT-labeled MBs with the micro-fluxgate sensor	Magnetic	Antibody/Glass	Serum	0.01–10 ng mL^−1^ LOD–0.01 ng mL^−1^	^64^
AuNPs immobilized on dithiol-modified surface	QCM	Antibody/Quartz	Serum	0.003–0.5 ng mL^−1^ LOD–0.0015 ng mL^−1^	^65^
CMOS-compatible SiNW array	Electrical	Antibody/SOI	PBS, Serum	0.000001–1 ng mL^−1^ LOD–1 fg mL^−1^ (PBS), LOD–30 fg mL^−1^ (Serum)	^18^
DEP assembled rGO based flexible aptasensor	Electrical	Aptamer/PET	PBS, Serum	0.001–10 ng mL^−1^ LOD–1.2 pg mL^−1^ (PBS) LOD–1.7 pg mL^−1^ (Serum)	**Present study**

Abs: antibodies, Al: aluminium, AuE: gold electrode, AuNPs: gold nanoparticles, cTnT: cardiac troponin T, CMOS: complementary metal-oxide semiconductor, CNTs: carbon nanotubes, CV: cyclic voltammetry, DEP: dielectrophoresis, DPV: differential pulse voltammetry, EIS: electrochemical impedance spectroscopy, GCE: glassy carbon electrode, LOD: limit of detection, MBs: magnetic beads, MI: molecular imprinting, MWCNTs: multiwalled carbon nanotubes, N-MIP: nano-molecularly imprinted polymer, PBS: phosphate buffer saline, PEI: polyethyleneimine, poly-*o*-ABA: poly-*o*-aminobenzoic acid, QCM: quartz crystal microbalance, rGO: reduced graphene oxide, SAM: self-assembled monolayer, SiNW: silicon nanowire, SPE: screen printed electrode, SPR: surface plasmon resonance, ZnO: zinc oxide.

Recently, flexible substrate-based sensors have become key components of portable, low-cost, and wearable devices for POCT applications. Furthermore, many researchers have worked to fabricate flexible electrodes for flexible substrates so that the sensors can be applied to any curved surfaces. In this respect, graphene is a good candidate because it provides a high degree of flexibility with high conductivity and transparency^21,22^. To date, several approaches such as mechanical exfoliation^23^, chemical vapour deposition (CVD)^24^, thermal annealing of SiC^25^, and the arc discharge method^26^ have been developed to produce single and multiple layers of graphene. Among them, CVD is an extensively used approach to produce large-area graphene sheets; however, it is a high-temperature process, hence restricting the substrate choice and making it costly to scale up the production of graphene sheets. In contrast, solution processing of graphene oxide (GO) is a simple, cost effective, and efficient way of preparing graphene-based thin films. Solution-processing techniques include membrane filtration^27,28^, dip coating^29^, layer-by-layer assembly^30,31^, spray coating^32^, and spin coating^33^. These facilitate the production of a large quantity of graphene that is compatible with various substrates such as silicon and plastic; however, deposition at specific locations is still a challenging issue, and it is difficult to maintain the uniformity and thickness of the thin films with these methods^34^.

Dielectrophoresis (DEP) is an electrical method for the deposition and manipulation of micrometer- and nanometer-sized particles dispersed in a liquid^35^, and it has been successfully applied to thermally expanded graphite oxide^36^, carbon nanotubes^37–39^, a few layers of graphene flakes and nanoribbons^40^, and a few layers (5–15) of reduced GO (rGO)^41^. DEP has many advantages, such as controlled deposition at specific locations, low cost, the ability to be performed at room temperature (RT), short deposition time (a few seconds), good thickness controllability, and good uniformity^40^. This contrasts with other deposition approaches such as dip coating and drop casting on pre-treated self-assembled monolayers (SAM), where it would take several hours to deposit graphene and it is difficult to maintain uniformity and deposition of graphene at predefined location^42,43^.

This study demonstrates a low-cost, flexible, and label-free electrical aptamer-based immunosensor (aptasensor) for cTnT detection using rGO sheets on a transparent substrate. GO was first deposited as a channel between source and drain electrodes using DEP onto a plastic [polyethylene terephthalate: (PET)] substrate with controlled alignment. The GO was then reduced to rGO using hydrazine vapour. The fabrication of aptasensors using DEP deposition of GO sheets followed by reduction of the sheets has never been explored before, although both aptasensors and rGO have attracted considerable attention recently. In fact, aptamers have shown numerous advantages compared to antibodies, including no variation in batch-to-batch process, easy modification, fast and low-cost production with high flexibility, stability and specificity, making them ideal candidates for POCT applications^44,45^. Moreover, the present DEP-assembly of GO and its subsequent reduction produced more stable rGO sheets than those directly deposited by DEP, leading to uniform and smooth deposition of rGO sheets as a thin film on the flexible substrate^46^. The present electrical aptasensor holds the following advantages: low-cost, simple, and rapid fabrication at room temperature along with high yield, high sensitivity, flexibility, and reusability.

The rGO channel modified with a linker of 1-pyrenebutanoic acid succinimidyl ester (PBSE) was further functionalized with a DNA aptamer against cTnT. The relative resistance change (RRC) of the aptasensor caused by the attachment of cTnT to the channel was quantified as the cTnT concentration in human serum (10-fold diluted) and phosphate buffered saline (PBS) varied from 1 pg mL^−1^ to 10 ng mL^−1^. The sensitivity, selectivity, reproducibility, and reusability of the developed aptasensor will be discussed.

## Results and Discussion

### Characterization of as-deposited GO and reduced GO sheets between Cr/Au electrodes

The Raman spectra of the GO and rGO sheets between Cr/Au electrodes are shown in Fig. S1A,B, respectively. The Raman spectrum of the DEP-deposited GO showed a dominant D band (at 1350 cm^−1^) and G band (at 1598 cm^−1^)^47^. The increased intensity of the D band for the rGO spectrum shows that sp^2^ structures of graphene or covalently attached functional groups were eliminated^36^.

The reduction of GO to rGO was also confirmed by XPS analysis (Fig. S2). The XPS data were collected from a thin film of GO and its reduced form rGO. The GO C1s XPS spectra demonstrate the bonding of carbon-oxygen functional groups such as C–C (284.6 eV), C–O (286.5 eV), C=O (287.6 eV), and O–C=O (288.9 eV)^48^. The atomic ratio of carbon to oxygen increased from ~1.8 to ~4.8 through reduction. The intensities of the C–O and C=O peaks also considerably reduced, and the O–C=O peak completely disappeared in the rGO spectra (Fig. S2). This was closely associated with an increase in the sp^2^/sp^3^ carbon peaks, showing that many oxygen-containing groups were eliminated and the most of the sp^2^ carbon networks were restored. The additional peak of C–N groups at 285.8 eV shows that some nitrogen atoms were introduced from the reducing agent hydrazine^41^.

**Figure 1:**
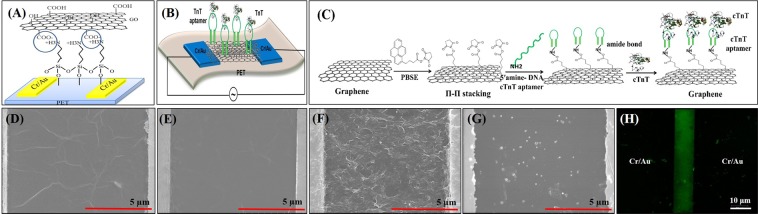
**(A)** A schematic of the DEP-deposition of GO sheets on an APTES-modified PET substrate. **(B)** Schematic of the rGO aptasensors for the detection of cTnT, which consisted of rGO sheets (DEP-deposition of GO and its subsequent reduction) and cTnT aptamers on an APTES-modified PET substrate. **(C)** Schematic of cTnT aptamer immobilization on the graphene surfaces via PBSE linker and cTnT aptamers. Field emission scanning electron microscopy (FE-SEM) images of DEP-assembled thin layers of GO sheets **(D)** before and **(E)** after reduction via hydrazine vapour between two Cr/Au electrodes, **(F)** direct DEP deposition of rGO sheets, and **(G)** the immobilized cTnT aptamers on the rGO surface. **(H)** Fluorescence image of the immobilized cTnT aptamers on the rGO surface.

Aptasensors with micrometer-sized channel lengths and widths (L: 10 µm and W: 100 µm) between two Cr/Au electrodes were fabricated using wet etching on a PET substrate (Fig. S3). FE-SEM images of the DEP-assembled GO sheets before and after hydrazine reduction are shown in Fig. 1D,E, respectively. The GO sheets showed a layered structure consisting of ultrathin and homogeneous films. Because of the folding of these films, it was possible to recognize the edges of individual GO sheets with crumpled areas. After reduction, the rGO sheets were very smooth (Fig. 1E). This contrasts with those directly deposited by DEP (Fig. 1F), which resulted in many folds and wrinkles between the Cr/Au electrodes, making it difficult to measure the thickness of the rGO sheets. Figure 1G shows an FE-SEM image of a cTnT DNA aptamer-modified rGO surface, with many tiny clusters on the rGO surface, demonstrating aggregates of cTnT aptamers^49^. Figure 1H shows a fluorescence image of FAM-modified cTnT aptamers on the rGO channel. The aptamers were selectively bound on the rGO surfaces via the PBSE linker, and the image clearly demonstrates that cTnT aptamers were deposited throughout the rGO sheets.

**Figure 2:**
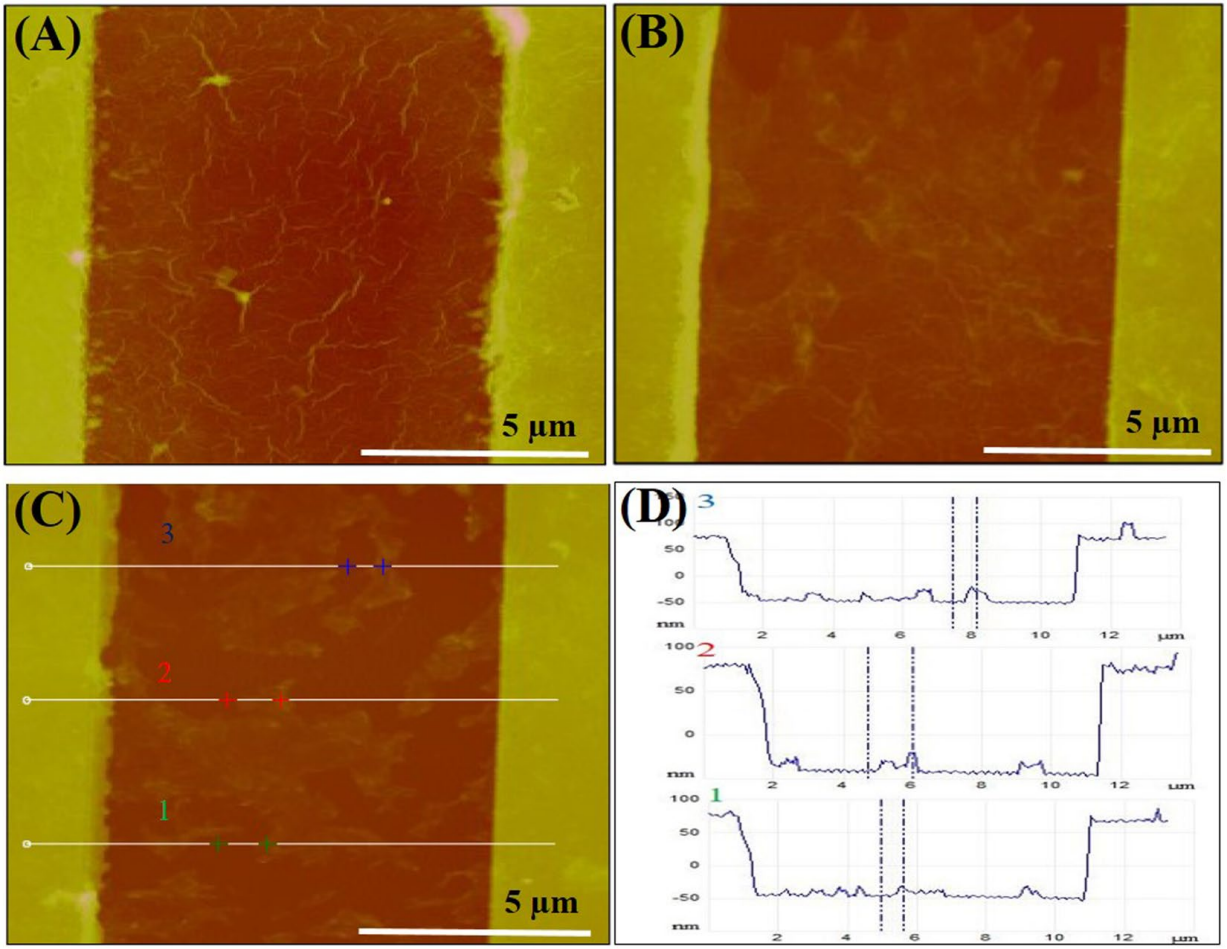
AFM images of DEP-deposited GO **(A)** and its reduced sheets (rGO) **(B)** between the two Cr/Au electrodes. **(C)** AFM image of the rGO surface. Lines 1, 2, and 3 represent the measuring lines between the Cr/Au electrodes. **(D)** Height profiles of the rGO sheets along the three lines in (C) measured by AFM.

AFM images of the DEP-assembled GO sheets before and after reduction are shown in Fig. 2A,B, respectively. The rGO sheets connected the Cr/Au electrodes with fewer wrinkles and folds than the GO sheets. Figures 2C,D show surface profiles of the rGO sheets along three lines between two Cr/Au electrodes. Based on the measured profiles, we observed several bumps in the rGO sheets along lines 1, 2, and 3, and the thicknesses of the rGO sheets were approximately 6.22, 16.01, and 12.87 nm, respectively. Therefore, the thickness of the rGO sheets inside the channel was estimated to be in the range of 6–16 nm.

A suspension of rGO sheets, which was synthesized through the same process except that GO sheets were reduced using hydrazine solution instead of hydrazine vapour, was also drop-cast onto a flat silicon surface, and AFM images of the rGO sheets and their corresponding profiles were analysed to determine the thickness of a single layer of rGO and hence the number of rGO layers deposited between the Cr/Au electrodes (Fig. S4). This was done because it was difficult to measure the thickness of a single rGO sheet between two Cr/Au electrodes when using DEP, despite the fact that the solution contained a large number of single-layered rGO sheets. The AFM measurements showed that the apparent thickness of the deposited single-layered rGO sheets varied from 1.2 nm to 1.5 nm. This is similar to the thickness of a single rGO layer reported in previous work^41^. These results demonstrate that most of the rGO sheets deposited by DEP were few-layered (4–10 layers). Moreover, the transmittance of a bare PET substrate and of rGO sheets between Cr/Au electrodes on a PET substrate was approximately 91% and 76%, respectively (Fig. S5).

Reduction of GO sheets using hydrazine solution did not etch the PET substrate and not harm the sensor characteristics. In contrast, hydrazine solution reacts with various types of materials including SiO_2_/Si substrate, with an etching rate (2 µm/min) on Si substrate^50^. This implies that a reduction process of GO sheets with hydrazine solution could be challenging for some device fabrication.

### Electrical measurements of the rGO aptasensors after each functionalization

To examine the electrical properties of the rGO aptasensor, the resistance of DEP-assembled GO was first measured, and it was on the order of several MΩ. After reduction of the GO sheets using hydrazine vapour and mild annealing at 150 °C for 2 h, the resistance of the sensors was measured to be 10–30 kΩ. The *I–V* characteristics of the DEP-assembled thin layers of GO sheets before and after reduction via hydrazine vapour between two Cr/Au electrodes on PET are shown in Fig. S6. The *I–V* characteristics of the aptasensors were also measured at each step of functionalization (Fig. S7). The *I–V* curves showed linear behaviour after each functionalization, showing good ohmic contact between the rGO sheets and the Cr/Au electrodes. The resistance of aptasensor increased after each functionalization: linker (PBSE), cTnT aptamer, blocking buffer (ethanolamine), and cTnT (1 pg mL^−1^). This result demonstrates that each binding event might change the electrical properties of the graphene sheets, causing reduction in charge-carrier density on the rGO surface. Moreover, binding of the lowest cTnT concentration (1 pg mL^−1^) on aptamer/rGO surface was observed through the change in *I*–*V* characteristics. This sufficient change in resistance shows the attachment of cTnT antigen on the rGO surface, and thus it may allow label-free detection of cTnT.

### Sensitivity and selectivity studies in PBS and human serum

**Figure 3:**
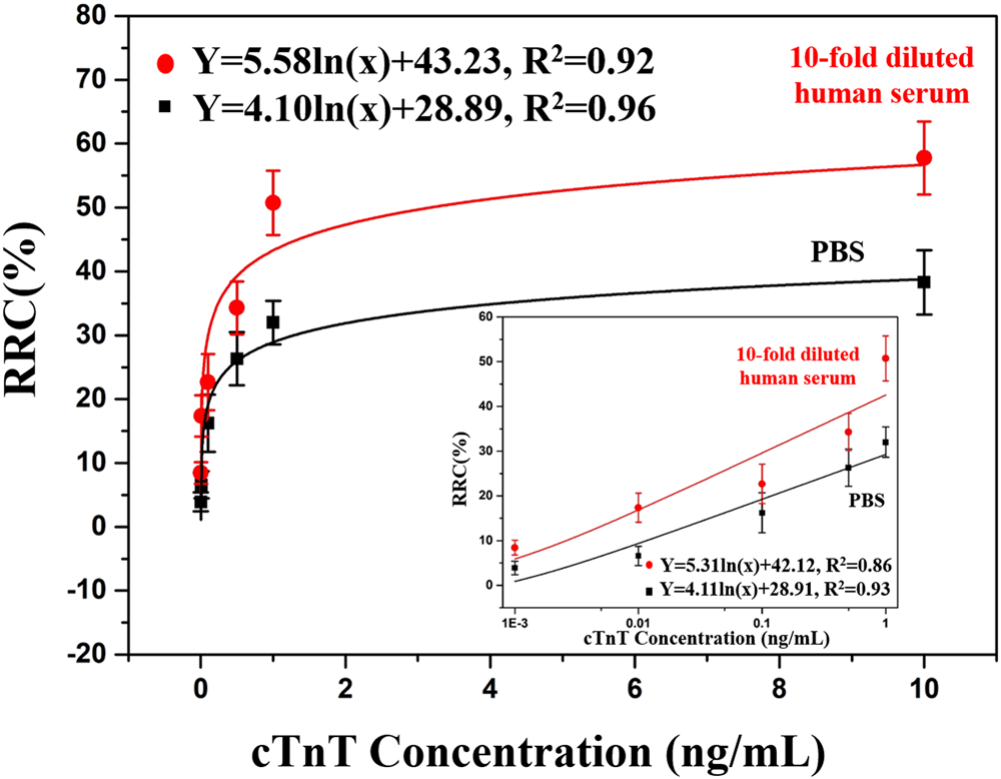
Relative resistance change (RRC) of the aptasensors as the concentration of cTnT in PBS (pH 7.4, 1×) and 10-fold-diluted human serum was varied from 1 pg mL^−1^ to 10 ng mL^−1^. The error bars indicate the standard deviations of the measurements. The inset shows the measurements from 1 pg mL^−1^ to 1 ng mL^−1^.

The sensitivity study of the rGO aptasensor was performed via measuring RRCs for various concentrations of cTnT. Figure 3 shows the RRCs of the rGO aptasensor with various concentrations of cTnT (1 pg mL^−1^–10 ng mL^−1^) in PBS (pH 7.4, 1×) and in human serum (10-fold diluted) in PBS (pH 7.4, 1×). The RRC increased significantly with increasing concentration of cTnT in both PBS and diluted human serum. This result shows that the specific cTnT aptamers fold as well-defined 3-D structure (loop formation) upon binding to complementary sequence of cTnT with high affinity^51^, allowing the change in RRC values. Moreover, cTnT protein adsorption on rGO sheets can affect drop-in conductance due to less electron*-*localization. Therefore, the modulation of the charge-carrier density might be a possible reason for change in resistance of the rGO sheets^52^. The developed aptasensor exhibited good fits [R^2^ = 0.96 for 1× PBS and 0.92 for human serum (10-fold diluted)] to the logarithm of cTnT concentrations (1 pg mL^−1^–10 ng mL^−1^) as compared to other immunosensors reported (Table 1). It should also be noted that RRCs in 1× PBS and 10-fold diluted human serum were shown to be linear (R^2^ = 93% and 86%, respectively) with respect to cTnT concentration from 1 pg mL^−1^ to 1 ng mL^−1^; however, as cTnT concentration increased more, RRCs started to be saturated. To support the electrical measurement, *I*–*V* curves for different concentrations of cTnT (1× PBS) were shown in supplementary information (Fig. S8)

Based on the interpolated graphs in Fig. 3 and the background noises, the detection limits for cTnT against the media (the signal-to-noise ratio = 3) were found to be 1.2 pg mL^−1^ for PBS (pH 7.4, 1×) and 1.7 pg mL^−1^ for human serum (10-fold diluted). Therefore, the present aptasensor is sensitive enough for clinical applications, considering that the detection limits of current ELISA kits are 10–30 pg mL^−1^ and the assay range is 10 pg mL^−1^–10 ng mL^−1^ according to the datasheets^53^.

**Figure 4:**
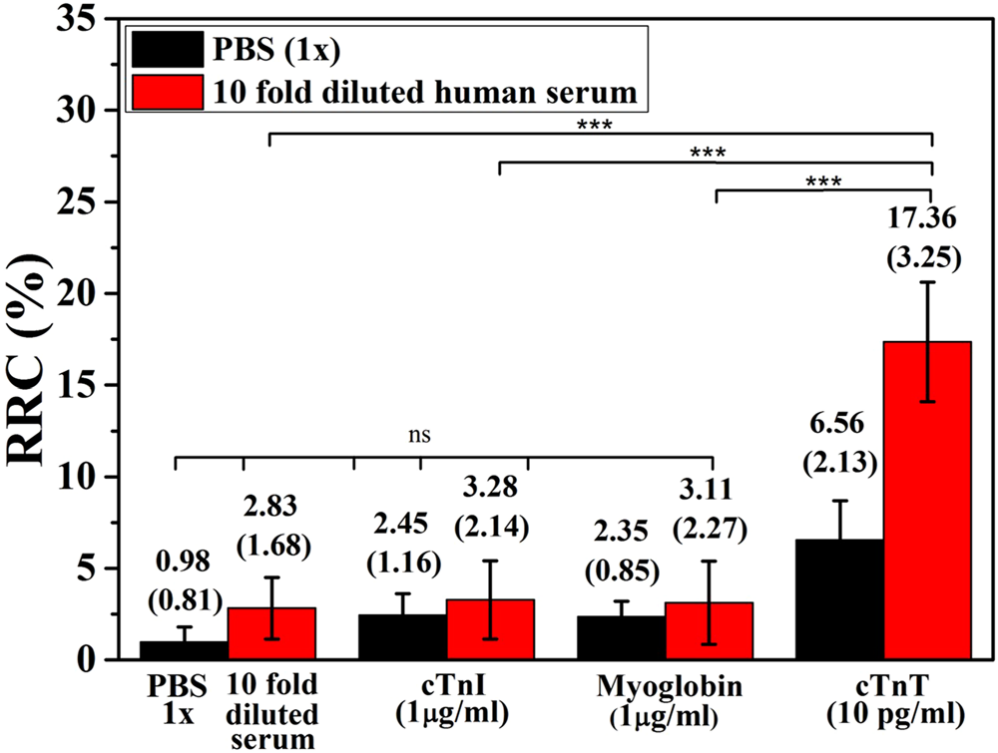
Selectivity test of the rGO aptasensors using PBS (pH 7.4, 1×), 10-fold-diluted human serum in PBS (pH 7.4, 1×), cTnI (1 µg mL^−1^), Myoglobin (1 µg mL^−1^) and cTnT (10 pg mL^−1^) in the two media. The error bars indicate the standard deviations of the measurements. One-way analysis of variance (ANOVA) was used to check whether the measured RRCs were significantly different. (ns: *p* > 0.05 and ***p < 0.0001).

To examine the selectivity of the proposed rGO aptasensor toward cTnT detection, the RRCs were measured with other cardiac biomarkers, troponin I and myoglobin, in PBS (pH 7.4, 1×), and human serum (10-fold diluted) (Fig. 4). When high-concentration (1 µg mL^−1^) troponin I and myoglobin in the two media were added to the sensors, a very small change in the RRCs was observed compared to the RRCs obtained for the media alone. However, when 10 pg mL^−1^ of cTnT was added to the sensors, the RRCs showed rapid and sharp increases. The RRCs of this cTnT concentration were significantly different from those of the two media alone, but there were no significant differences between other cardiac biomarkers (cTnI and myoglobin) and the media alone. These results confirm that the developed rGO aptasensor has high selectivity toward cTnT.

### Reusability and reproducibility of the aptasensor

**Figure 5:**
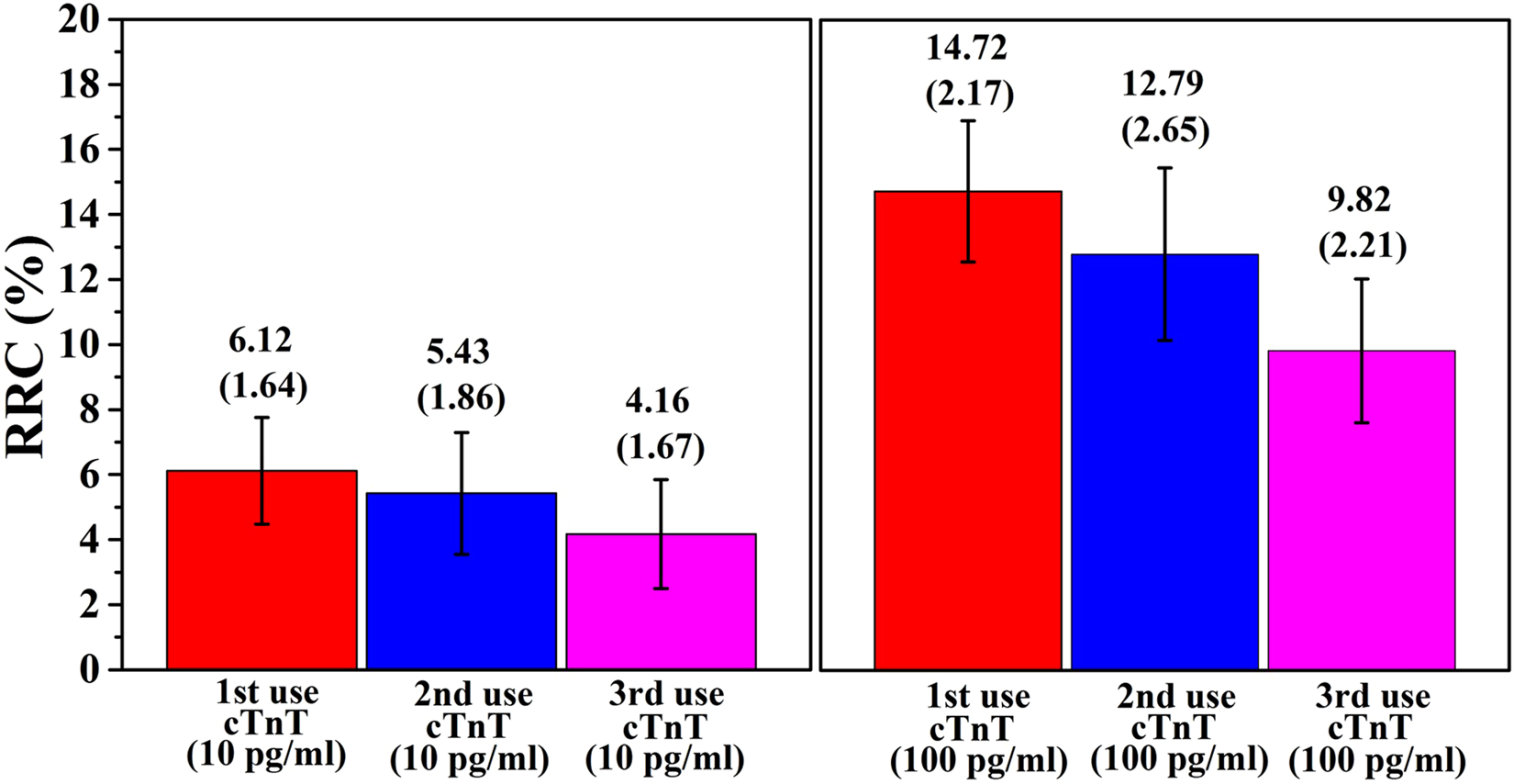
Reusability of the aptasensor. Relative resistance change (RRC) of the aptasensors at two concentrations of cTnT (10 pg mL^−1^ to 100 pg mL^−1^) in PBS (pH 7.4, 1×). The error bars indicate the standard deviations of the measurements.

Figure 5 shows RRC (Δ*R* = (*R − R*_0_*)/R*_0_) with recycling of the aptasensors, where *R* is the resistance of either new or reused aptasensor after cTnT immobilization, and *R*_0_ is the resistance of aptasensor after immobilization with cTnT aptamers and a blocking agent. The resistance was degraded consistently as the number of uses increased during the first three repeated uses owing to aptamer degradation.

To determine the reproducibility of the aptasensors, five samples (100 pg mL^−1^ in 10-fold diluted human serum) were evaluated on the same day and then stored at 4 °C for one week (Table S1). The relative standard deviation of the RRC values was measured to be 7.24% within 1 day and 11.29% after 1 week, demonstrating that the RRC values were consistent within and between the batches.

We also conducted another test on the adhesion of the fabricated rGO sheets. The resistance changes of the present rGO sensors (DEP deposition of GO and its reduction) and those of the sensors fabricated using direct deposition of rGO by DEP were measured with the number of times of a washing and nitrogen drying treatment (Table 2). The average RRC of the present sensors between the first and the fourth treatments was 4.2%, whereas that of the sensors using the direct deposition of rGO by DEP was 64.5%. This result indicates that the present rGO sensors using the DEP deposition of GO and its subsequent reduction along with APTES functionalization showed better stability than the direct DEP deposition of rGO sheets.

**Table 2:** The comparison between two treatments with the number of times of washing and drying process.

	1^st^ gently washing with DI water and then N_2_ dry	2^nd^ gently washing with DI water and then N_2_ dry	3^rd^ gently washing with DI water and then N_2_ dry	4^th^ gently washing with DI water and then N_2_ dry	Relative resistance change (1^st^ – 4^th^) (%)
(Top) Average: 64.58%	8.91 kΩ	9.50 kΩ	10.24 kΩ	13.78 kΩ	54.65
	4.57 kΩ	6.11 kΩ	7.00 kΩ	9.15 kΩ	108.0
	6.50 kΩ	7.61 kΩ	8.11 kΩ	11.45 kΩ	76.15
	5.45 kΩ	6.34 kΩ	7.56 kΩ	7.71 kΩ	41.46
	7.87 kΩ	8.40 kΩ	10.05 kΩ	11.22 kΩ	42.56
(Bottom) Average: 4.24%	15.82 kΩ	15.80 kΩ	15.98 kΩ	16.41kΩ	3.72
	19.54 kΩ	20.14 kΩ	20.30 kΩ	20.85 kΩ	6.70
	14.78 kΩ	14.90 kΩ	14.98 kΩ	15.03 kΩ	1.69
	11.21 kΩ	11.32 kΩ	11.85 kΩ	11.90 kΩ	6.15
	17.60 kΩ	16.98 kΩ	17.50 kΩ	17.10 kΩ	2.92

(Top) Direct deposition of rGO sheets by DEP on a bare PET substrate, and then heating at 150 °C/2 h. (Bottom) Direct deposition of GO sheets by DEP on an APTES-coated PET substrate, their reduction (rGO), and then heating at 150 °C/2 h.

## Conclusions

We have demonstrated a low-cost, flexible, highly sensitive and label-free electrical aptasensors using DEP-deposited GO and its subsequent reduction to detect the cardiac biomarker troponin T. A few layers (4–10 layers) of GO were deposited between two predefined Cr/Au electrodes on an APTES-modified, flexible and transparent PET substrate by DEP and were reduced to rGO using hydrazine vapour, which did not damage PET substrates unlike silicon substrates. In terms of fabricating an rGO channel, DEP deposition of GO and its reduction was better than the direct DEP deposition of rGO, demonstrating the uniform and robust deposition of both GO and its reduced form, rGO, on the PET substrates. Moreover, the DEP process used in this study provided a very high yield, and deposition was controlled to a specific location at room temperature within a short time (a few seconds), contrasting with other deposition approaches such as drop casting and CVD.

## Materials and Methods

### Materials and reagents

Graphite (282863), H_2_SO_4_ (339741), KMnO_4_ (223468), H_2_O_2_ (216763), HCl (H1758), NaNO_3_ (S5506), 3-aminopropyltriethoxylsilane (APTES) solution (440140), hydrazine solution (35%) (309400), ethanolamine (NH_2_CH_2_CH_2_OH; ≥98%) (E9508), Cr etchant (651826), Au etchant (651818), and myoglobin (MB; M0630) were purchased from Sigma-Aldrich (USA); 5′-amine modified, and 5′-amine/3′-FAM (6-carboxy fluorescein) modified cTnT aptamers (091), where the sequence of the aptamers was 5′-ATACGGGAGCCAACACCAGGACTAACATTATAAGAATTGCGAATAATCATTGGAGAGCAGGTGTGACGGAT-3′, were obtained from OTC Biotech (USA); cTnT (9202–1107) and cTnI (9202–0707) antigens were obtained from AbD-Serotec (USA); normal human serum (S1-100ML) was procured from Merck Millipore (USA); diethylpyrocarbonate (DEPC)-treated water (W2004) and dimethylformamide (DMF) (D1021; 98%) were procured from Biosesang (South Korea); PBSE (P130) was procured from Thermo Scientific (USA); and PBS (pH 7.4, 10×) was procured from Life Technologies (South Korea). DEPC-treated water was used for dilution of the aptamer stock solution, which was stored at −20 °C before use. Deionized (DI) water (18.2 MΩ) was prepared from the water purification system (Millipore).

### GO synthesis

GO was obtained using a modified Hummer’s method^54,55^. In brief, graphite powder (1 g) and NaNO_3_ (0.5 g) were mixed together, and concentrated H_2_SO_4_ acid (23 ml, 99.99%) was carefully added under stirring at 600 rpm (10 s^−1^) for 1 h. In addition, KMnO_4_ (3 g) was added slowly to the solution on a stirrer plate at 600 rpm, and the solution temperature should be below 20 °C to avoid overheating and an explosion. Then, the solution was stirred on a stirrer plate at 600 rpm and 35 °C for 2 h. The resulting solution was mixed with DI water (50 mL) under stirring at 1200 rpm (20 s^−1^), resulting in a dark brown suspension. To make the completion oxidation with KMnO_4_, the resulting suspension was then mixed with 5 mL of a 30% H_2_O_2_ solution, and then DI water (100 mL) was added. The solution was treated with 10% HCl and then with DI water, followed by centrifugation at 5000 rpm (83 s^−1^). This washing step was repeated four times for 60 min each. This GO suspension was poured onto a glass dish and left to dry in an oven at 80 °C for 24 h, leading to GO powders.

### Fabrication of two electrodes and DEP assembly of GO sheets

Two electrodes were fabricated on a PET substrate using photolithography and a wet etching process. First, a sheet of PET (thickness: 250 µm, diameter: 100 mm) was cleaned in acetone, methanol, and isopropyl alcohol consecutively for 5 min each. The substrate was then rinsed with DI water for 1 min and dried with nitrogen (N_2_) gas. Deposition of Cr and Au (thicknesses: 10 nm and 100 nm, respectively) were made consecutively using electron beam evaporation. The electrodes were then fabricated by applying Au and Cr etchants consecutively before removing the protective photoresist. The gap between these two electrodes was 10 µm, and the width of the facing sides of the two electrodes was 100 µm. Finally, this substrate was cut into immunosensor chips, each measuring 10 × 10 mm^2^. After the chips were treated with oxygen plasma for 10 min with a source power of 200 W, APTES solution (2 wt.%) in ethanol was pipetted onto the channel area and incubated at RT for 30 min^56^. The chips were then rinsed with DI water and dried with a N_2_ gas stream.

GO suspension was made by suspending the synthesized GO powder in DI water (10 µg mL^−1^) by using 60 min sonication (Bransonic, 5510) to remove any aggregates, centrifuging the solution at 5000 rpm (83 s^−1^) for 30 min, and then collecting the supernatant. After pipetting 10 µL of the collected GO suspension between the predefined Cr/Au electrodes coated with APTES, providing additional adhesion between PET and GO surfaces, a thin film of GO sheets was created by supplying 10 Vpp (peak-to-peak) at 500 kHz for 30 s (Fig. 1A).

### Reduction of GO sheets on a PET substrate

The DEP-deposited GO film on a PET substrate was reduced to rGO by treating with hydrazine vapour. First, the purchased hydrazine solution was diluted with DI water to make 20 mL of a 15% (v/v) hydrazine solution. The chips with GO were moved into a small glass petri dish, which was kept inside a larger glass petri dish containing the hydrazine solution. The larger dish was covered with a glass lid and a parafilm tape, and kept on a hot plate at 90 °C for 18 h^48^. Afterwards, the dish was removed and the chips were thoroughly rinsed with DI water. After this reduction step, the chips were immediately placed in a vacuum oven at 150 °C for 2 h, in order to remove the residue and enhance the adhesion between the rGO sheets and the electrodes.

### Channel functionalization, resistance measurements, and reusability test

The chips were thoroughly rinsed with DI water, dried with N_2_ gas, and then incubated with 5 mM of PBSE (linker) in DMF at RT for 1 h, followed by treating with DMF and DI water consecutively. PBSE provides pyrene groups for π–π interactions to the planes of rGO sheets, leading to stable noncovalent interaction via π-electron donating and accepting, and its terminal functional group remains free, allowing covalent attachment of biomolecules due to their amine groups. The chips were then incubated in 5′-amine–modified DNA cTnT aptamers (10 µg mL^−1^) in DEPC-treated water at 37 °C for 2 h, thoroughly rinsed with PBS (pH 7.4, 1×) to remove loosely attached aptamers, and then air-dried at RT for 5 min. The cTnT aptamer was covalently attached via binding of −NH_2_ groups with succinimide ester generated on the graphene surface. The chips were then incubated in ethanolamine (100 mM) at RT for 1 h to preclude possible non-specific binding on the rGO surface, and then rinsed with PBS (pH 7.4, 1×). cTnT (10 ng mL^−1^ to 1 pg mL^−1^) in PBS (pH 7.4, 1×) and in human serum (10-fold diluted) with PBS (pH 7.4, 1×) was applied to measure the RRC of the aptasensors (Fig. 1B,C). The rGO surfaces were also incubated in 5′-amine and 3′-FAM-modified cTnT aptamers with the same sequence used for cTnT detection (10 µg mL^−1^) diluted in DEPC-treated water at 37 °C for 2 h, followed by rinsing with PBS (pH 7.4, 1×), and air-dried at RT, to obtain a fluorescence image of the aptamers deposited on the channels. Figure 1B shows a diagram of the rGO aptasensor for the detection of cTnT.

RRC is defined as Δ*R* = (*R* − *R*_0_)/*R*_0_, where *R* is the resistance of aptasensor after cTnT immobilization, and *R*_0_ is the resistance of aptasensor after immobilization with the cTnT aptamer and blocking agent. All electrical measurements were performed with a Keithley 2636B source meter. The current–voltage (*I–V)* measurements of the rGO aptasensors were performed from −2.0 V to + 2.0 V at each step of functionalization on the rGO surface.

The reusability of the aptasensors was evaluated, and the tests consisted of the following steps: the immobilization of cTnT (100 and 10 pg mL^−1^) in 1× PBS on the aptasensors followed by rinsing with PBS and resistance measurements, and the addition of a 10% NaCl for 15 min, followed by rinsing with DEPC-treated water, dissociating the cTnT-aptamer complexes without detaching the aptamer^57^.

### Characterization of rGO aptasensor

The DEP-deposited GO sheets and their reduced sheets (rGO) were characterized by a tapping mode atomic force microscope (Bruker Instrument, USA) and Raman spectroscopy (300R, WITec, Germany). A field emission scanning electron microscope (FE-SEM; S-4800, Hitachi, Japan) was used to image the GO and rGO sheets between the two electrodes. The samples were platinum-coated with ion sputtering (E-1045, Hitachi, Japan) before FE-SEM imaging. The chemical bonding of the synthesized GO and rGO sheets were studied by X-ray photoelectron spectroscopy (XPS; K-Alpha, Thermo Scientific, USA). Optical transmission of the aptasensors was estimated with ultraviolet–visible–near-infrared spectroscopy (UV–vis–NIR; Cary 5000, Agilent, USA). Optical images were also obtained with an optical microscope (Eclipse 80i, Nikon, Japan) and a charge-coupled device (CCD) camera (CoolSNAP HQ2 Monochrome, Photometrics, USA). Fluorescence images were taken using a multiphoton confocal laser scanning microscope (ZEISS LSM780 NLO, Germany).

### Statistical analysis

Each experiment was analysed with four aptasensors (*n* = 5 replicates). The averaged values with their standard deviations (indicated as error bars) are shown in the figures. One-way analysis of variance (ANOVA) was used to determine whether the measured RRCs were significantly different between cTnT concentrations, and they were counted significantly different for *p* < 0.05.

## Supplementary information

SUPPLEMENTARY INFO
