# Supplementary material for: Flexible electrical aptasensor using dielectrophoretic assembly of graphene oxide and its subsequent reduction for cardiac biomarker detection
Source: Sci Rep. 2019 Apr 12;9:5970. doi: 10.1038/s41598-019-42506-1 (PMC6461687; doi:10.1038/s41598-019-42506-1)
Supplement: Supplementary file 1 — SUPPLEMENTARY INFO [file 41598_2019_42506_MOESM1_ESM.pdf]

Supplementary Information

Flexible electrical aptasensor using dielectrophoretic assembly of graphene oxide and its subsequent reduction for cardiac biomarker detection

Abhinav Sharma[a], Jaesung Jang[b,c,†]

[a] School of Materials Science and Engineering, Ulsan National Institute of Science and Technology (UNIST), Ulsan 44919, Republic of Korea

[b] School of Mechanical, Aerospace and Nuclear Engineering, UNIST, Ulsan 44919, Republic of Korea

[c] Department of Biomedical Engineering, UNIST, Ulsan 44919, Republic of Korea

[†]Correspondence should be addressed to jjang@unist.ac.kr; Tel: +82-52-217-2323; Fax: +82-52-217-2449

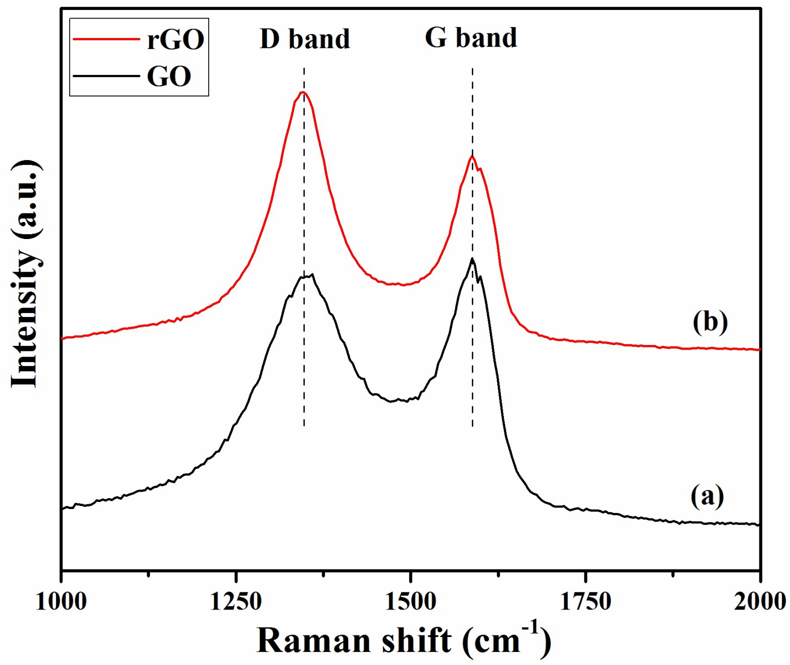

Fig. S1. Raman spectra of (a) DEP-deposited GO and (b) its reduced GO.

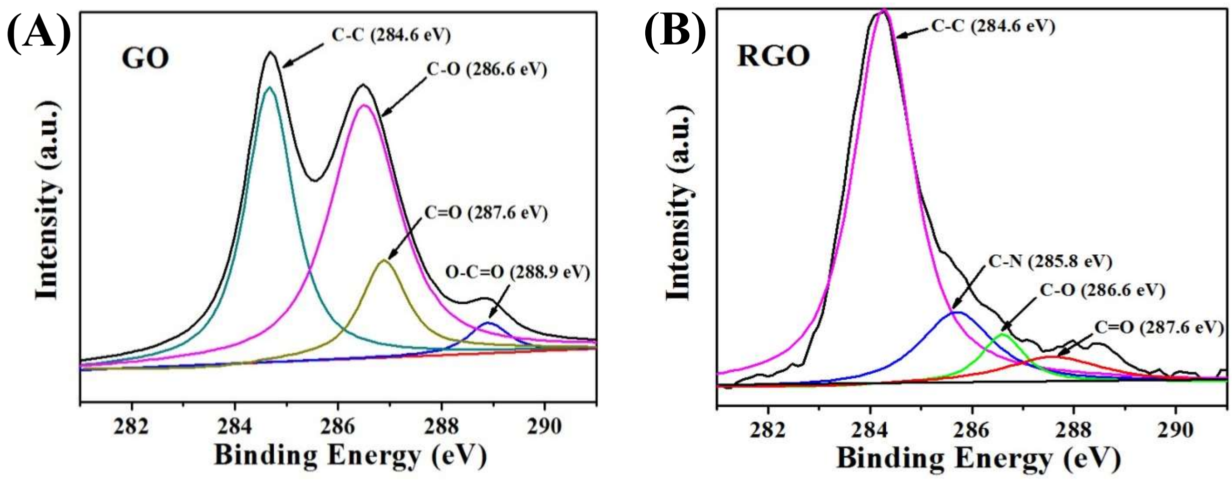

Fig. S2. (A) XPS data of GO sheets DEP-deposited on a PET substrate, and (B) XPS data of reduced GO via hydrazine vapour on a PET substrate.

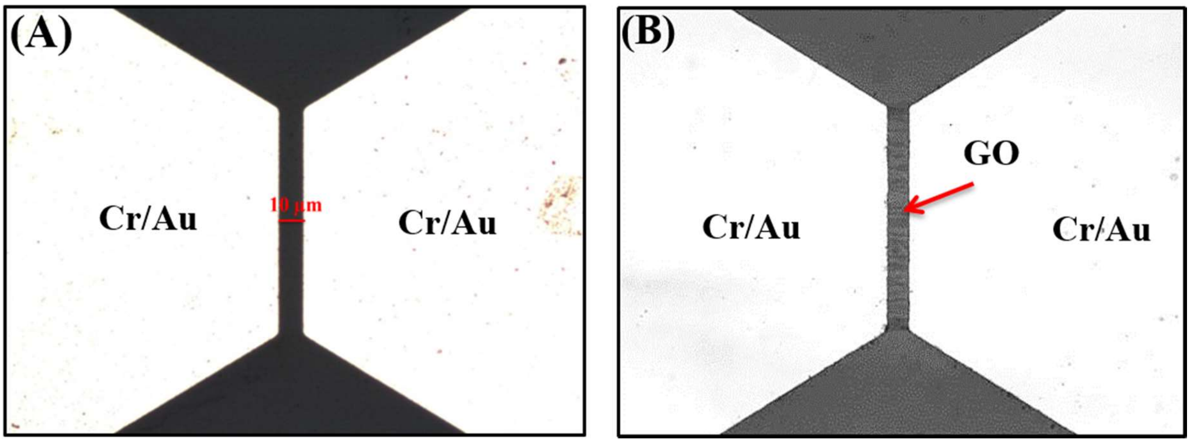

Fig. S3. (A) Optical image of patterned electrodes (Cr/Au) (L – 10 µm, W – 100 µm) on PET substrate, and (B) Optical image of deposited GO sheets between Cr/Au electrodes by DEP.

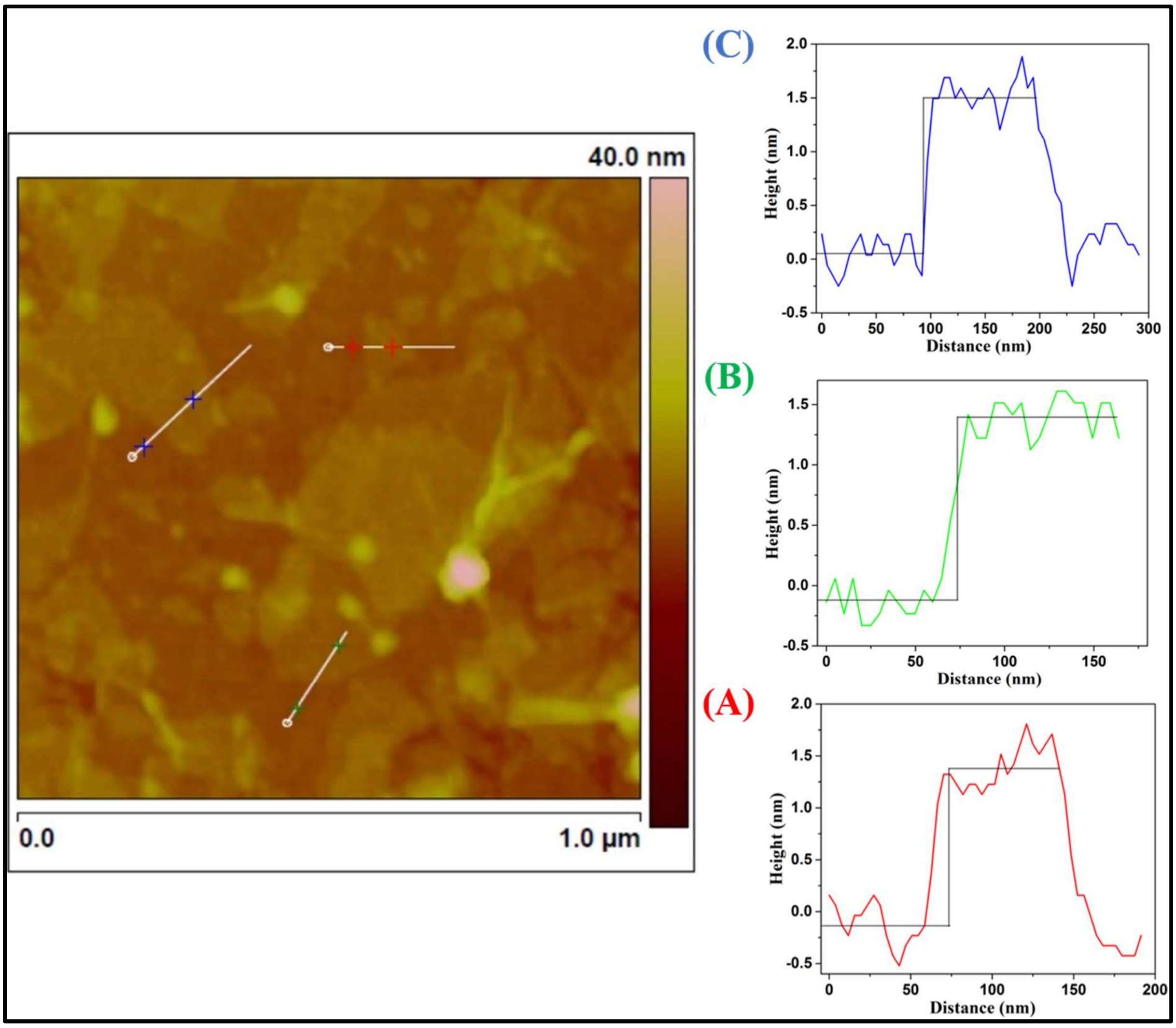

Fig. S4. Tapping-mode AFM images of rGO sheets drop-cast on a flat silicon surface with height profiles indicating that the majority of the sheets are single layered. The thickness varied from 1.2 nm to 1.5 nm. The step heights along the lines show 1.2 ± 0.2 nm (A), 1.4 ± 0.2 nm (B), and 1.5 ± 0.2 nm (C).

Transparency measurement

We measured UV-visible spectra of a bare PET substrate and rGO sheets between Cr/Au electrodes on a PET substrate at 550 nm. The optical transmittance spectra of these rGO sensors exhibited transparency (~76%) in the wavelength range of 300–700 nm (visible light) while the bare PET substrate showed slightly higher transparency (~91%). The inset shows a photograph of the transparent and flexible rGO based immunosensor. Transparent, and flexible biosensors are considered an attractive sensing platform because they allow both optical and electrical measurements in various experimental set-ups for biosensing and bio-MEMS.

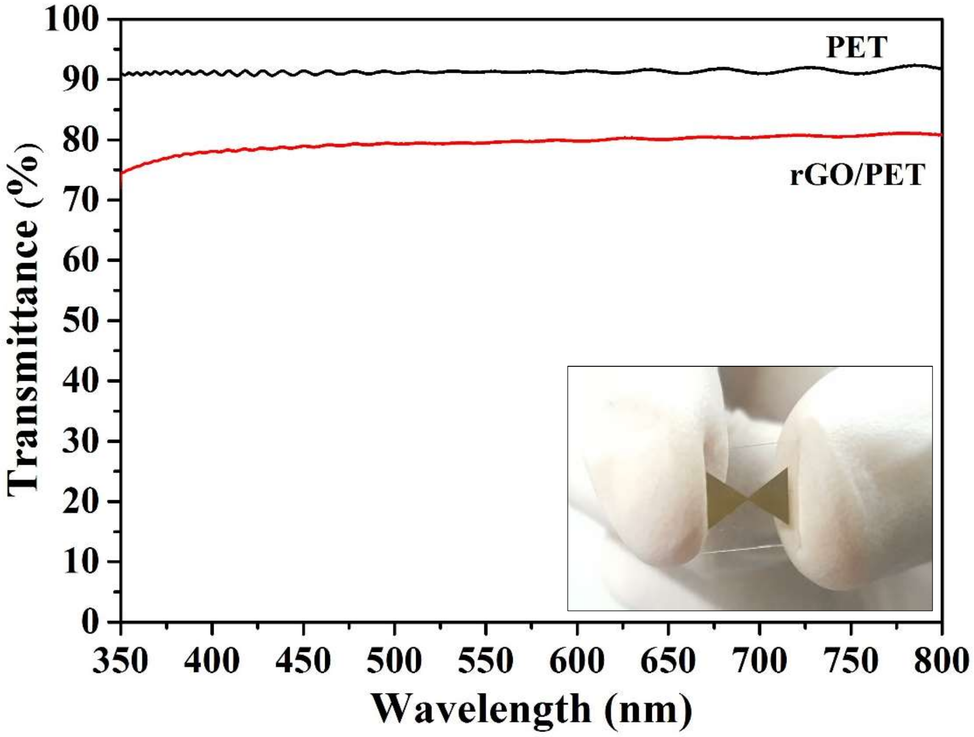

Fig. S5. Optical transmittance of a bare PET substrate, and DEP-assembled rGO transparent aptasensor on PET substrate. The inset shows a photograph of the transparent, and flexible rGO sheets between the electrodes (Cr/Au).

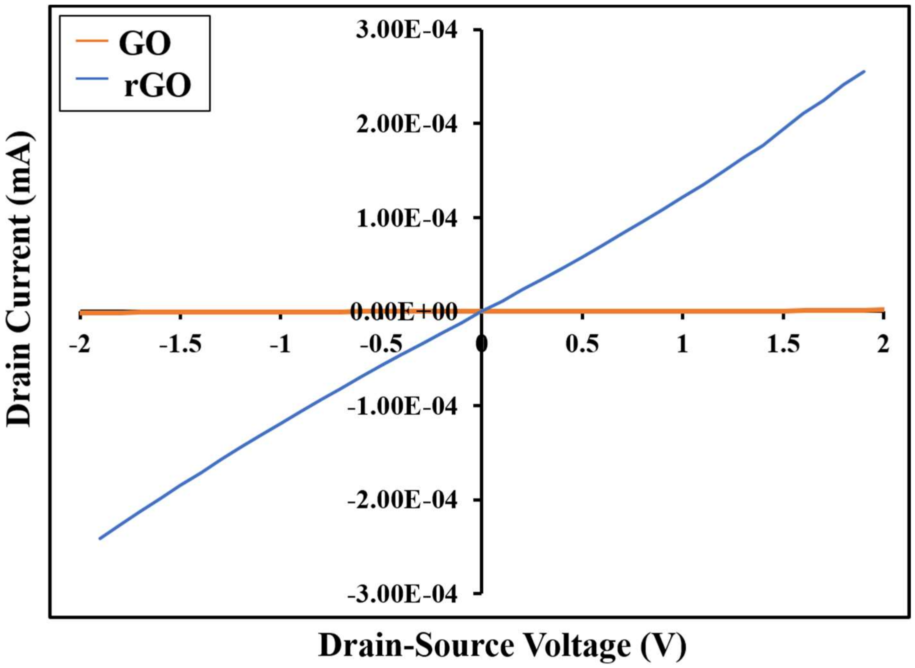

Fig. S6. *I-V* measurements of the thin layers of GO and rGO sheets assembled between two Cr/Au electrodes on PET.

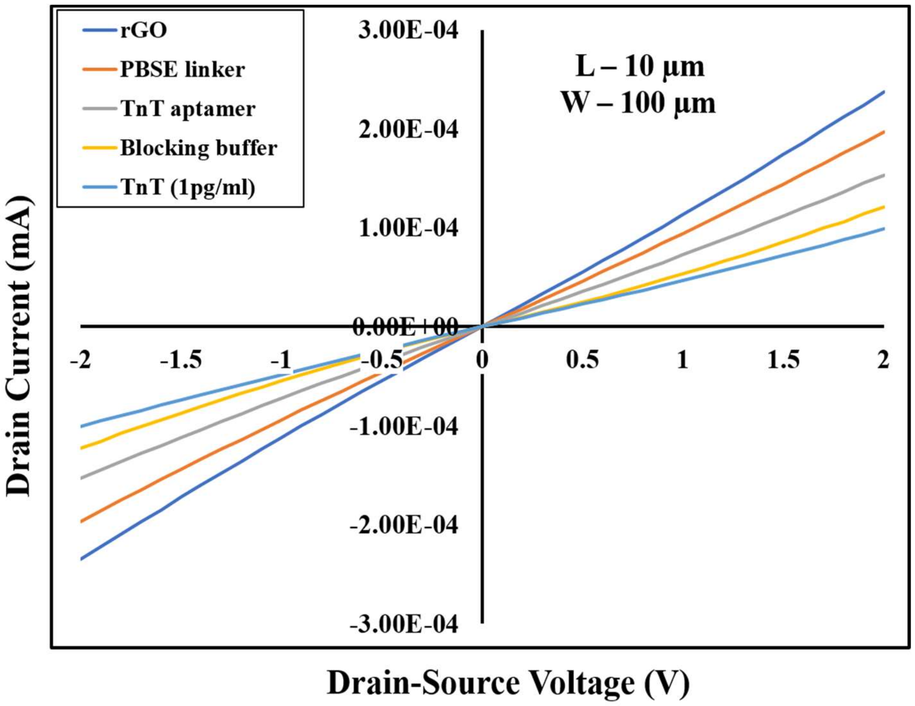

Fig. S7. *I-V* measurements of the rGO aptasensor (L: 10 µm and W: 100 µm) modified linker (PBSE), cTnT aptamer, blocking buffer (ethanolamine) and cTnT antigen (1pg/mL).

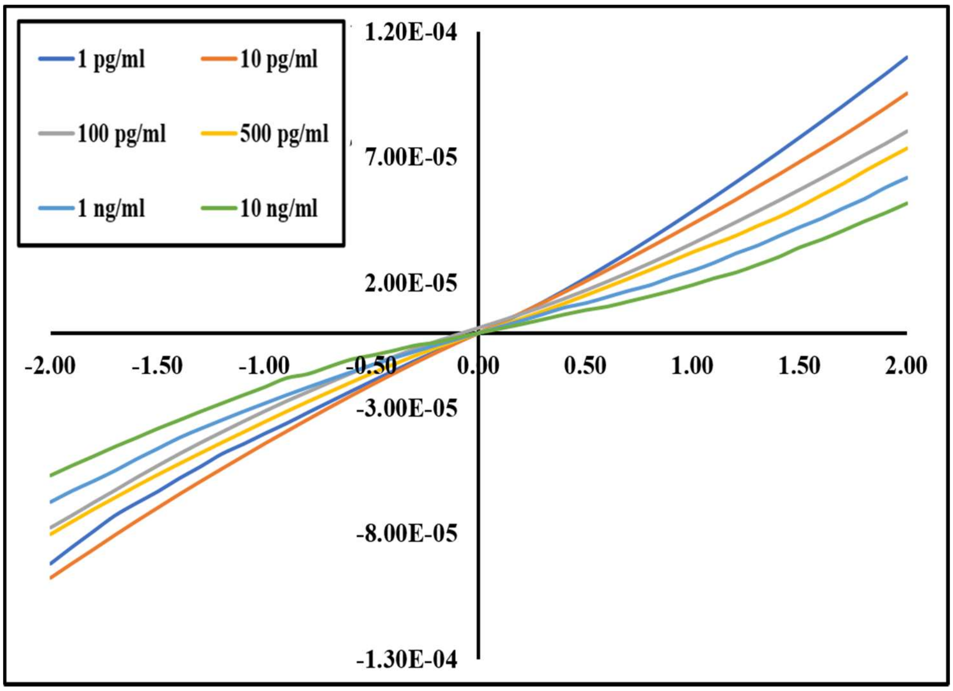

Fig. S8. *I-V* curves of the aptasensor for different concentrations of cTnT (1x PBS).

Table S1. Reproducibility tests of the aptasensors

cTnT(100 pg mL^{-1}) 10-fold-diluted human serum	1	2	3	4	5	Standard deviation (SD)	Mean value	*Relative standard deviation (RSD %) (n=5)
1st day	26.34	29.49	25.72	24.21	26.76	1.92	26.50	7.24 %
7th day	29.02	33.99	26.68	25.95	31.36	3.32	29.40	11.29 %

***Relative standard deviation (RSD %) = (SD/Mean) × 100**
